# MicroR-542-3p can mediate *ILK* and further inhibit cell proliferation, migration and invasion in osteosarcoma cells

**DOI:** 10.18632/aging.101698

**Published:** 2019-01-11

**Authors:** Wei Cai, Yong Xu, Wenshan Zuo, Zhen Su

**Affiliations:** 1 Department of Orthopedics, Huai’An First People’s Hospital, Nanjing Medical University, Huai’an 223300, Jiangsu, China; 2 Department of Anesthesiology, Huai’An First People’s Hospital, Nanjing Medical University, Huai’an 223300, Jiangsu, China

**Keywords:** cognitive MiR-542-3p, ILK, osteosarcoma, cell proliferation, migration, invasion

## Abstract

MiR-542-3p and its target gene integrin linked kinase (*ILK*) in human osteosarcoma together with the differentially expressed genes from osteosarcoma tissues was analyzed through bioinformatics analysis in this study. Real time quantitative polymerase chain reaction (qRT-PCR) and western blot showed that the miR-542-3p expression decreased while the *ILK* expression increased in the osteosarcoma tissues. The overexpressed miR-542-3p or silenced *ILK* restrained cell invasion, proliferation and migration and arrested cell cycle, facilitated cell apoptosis in U-2OS and 143B cells. The dual-luciferase assay confirmed the targeting relationship between miR-542-3p and *ILK*. MiR-542-3p overexpression inhibited osteosarcoma growth *in vivo*. In conclusion, miR-542-3p overexpression down-regulated its target gene *ILK*, promoted osteosarcoma cells apoptosis and inhibited their proliferation, migration and invasion.

## Introduction

Osteosarcoma is the first leading cause for sarcoma-related deaths in youngsters and children [1-3]. It happens mainly around body areas with active bone growth and reparation, such as the metaphysis of long bones of limbs [4]. Initially, the therapeutic method of osteosarcoma primarily includes surgical amputation, which may result in high risk of morbidity, trauma and low long-term survival rate. Over the past decade, advances in osteosarcoma therapy have improved patient outcomes, dramatically increasing the five-year survival rate of osteosarcoma patients to approximately 60-70% [4-6]. Despite the significant contributions made in surgery and neo-adjuvant chemotherapy, the clinical outcomes and prognosis have made little progress in the past decades [7]. At present, we’re still not clear about the accurate mechanism about the progression and development of osteosarcoma. Thus, verifying molecular mechanism of osteosarcoma development is of vital significance for diagnosis and treatment [4,8].

MicroRNAs (miRNAs) are small non-coding, single stranded RNAs which control the vital biological processes, such as angiogenesis, cell differentiation, tumorigenesis, apoptosis, proliferation, and migration by suppressing gene expression after transcription [3]. An increasing number of evidence has shown that miRNAs were involved in various types of cancers. Dysregulation of miRNA was related with the pathogenesis of various kinds of cancers, including osteosarcoma [5]. MiRNAs may serve as tumor inhibitors or oncogenes, hinging on whether they precisely target the tumor‑suppressor genes or oncogenes [9]. Each miRNA is predicted hundreds of genes, and each transcript may interact with multiple miRNAs in turn [10]. The miRNA-542-3p (miR-542-3p), located in Xq26.3, has been verified as a tumor inhibitor gene in plenty of cancers. Previous studies had validated that it was down-regulated in various type of human malignancies, including bladder cancer, astrocytoma, and esophageal squamous cell carcinoma [11,12]. Recent researches have revealed that miR-542-3p is relevant with tumor progression through c-Src-related oncogenic pathways. MiR-542-3p, nevertheless, causes growth arrest while inhibits tumor angiogenesis by targeting angiopoietin-2 [9] *ILK* is also a target gene of miR-542-3p, and previous studies had proved that *ILK* overexpression is related to the survival and recurrence of oral squamous cell carcinoma patients [13]. Oneyama et al. revealed that c-Src–miR-542-3p–*ILK*–FAK circuit played a crucial role in controlling tumor progression. Furthermore, *ILK* was identified as a conserved target of miR-542-3p [14].

*Integrin-linked kinase* (*ILK*) is a serine/threonine protein kinase located in focal adhesions, which promotes cell growth, proliferation, differentiation, migration and invasion [15]. *ILK* expression and activity have been revealed to be increased in association with the tumor grade, T status, lymph node metastasis and survival rate of lung cancer patients [16]. In some cancers, however, *ILK* is often overexpressed, leading to the increased cancer growth with cell proliferation, migration, and epithelial-mesenchymal transition [17]. Furthermore, *ILK* has been validated to accelerate cancer cell invasion and migration by giving rise to EMT process [18]. The overexpression of *ILK* has also been discovered to facilitate glioma cell invasion and migration and down-regulate E-cadherin. Besides, the inhibition of *ILK* has been shown to lead to cell cycle stagnation and stimulate apoptosis in PTEN-negative prostate cancer cells [15]. Indeed, previous studies have demonstrated that the increased *ILK* expression in poorly differentiated thyroid cancer and confirmed the relationship between *ILK* overexpression and poor prognosis [17].

In this study, the influence of miR-542-3p and its target gene *ILK* on human osteosarcoma was observed. MTT assay, flow cytometry, wound healing assay, transwell and plate clone formation assay were adopted to validate the migration, apoptosis and proliferation of osteosarcoma. Then we conducted the nude mouse transplantation tumor experiment to further analyze the influence of miR-542-3p and *ILK* on osteosarcoma, which may provide novelty insights into the treatment for osteosarcoma.

## RESULTS

### MiR-542-3p was down-regulated in osteosarcoma cells and tissues

**Figure 1:**
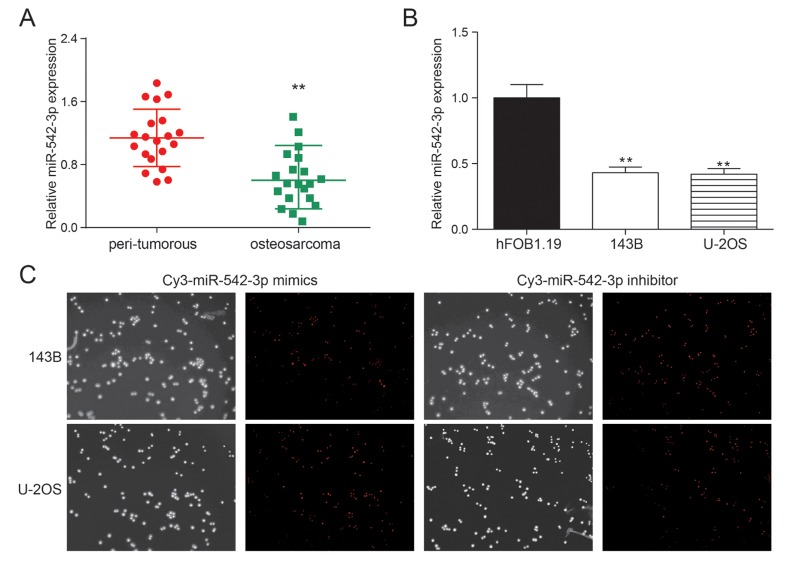
**miR-542-3p was down-regulated in osteosarcoma tissues and cells.** (**A**) The results of qRT-PCR showed that miR-542-3p was low expressed in osteosarcoma tissues. **P<0.01 compared to the peri-tumorous group; (**B**) The expression of miR-542-3p in cell lines 143B and U-2OS were lower than that in normal human osteoblastic cell line hFOB1.19. **P<0.01 compared to the hFOB1.19 group; (**C**) The miR-542-3p mimics and inhibitor had high transfection efficiency.

The expression of miR-542-3p in 20 pairs of osteosarcoma tissue samples were detected by qRT-PCR. The expression of miR-542-3p was remarkably down-regulated in osteosarcoma tissues compared with the nearby tissues (*P*<0.01, Figure 1A). The expression of miR-542-3p was detected in osteosarcoma cell lines 143B, U-2OS and normal human osteoblasts hFOB.19. The expression of miR-542-3p was noticeably lower in comparison with normal cells in osteosarcoma cells (*P*<0.01, Figure 1B). Figure 1C showed the transfection efficiency of miR-542-3p mimics and inhibitor with high transfection efficiency.

### MiR-542-3p restrained the proliferation of osteosarcoma cells

**Figure 2:**
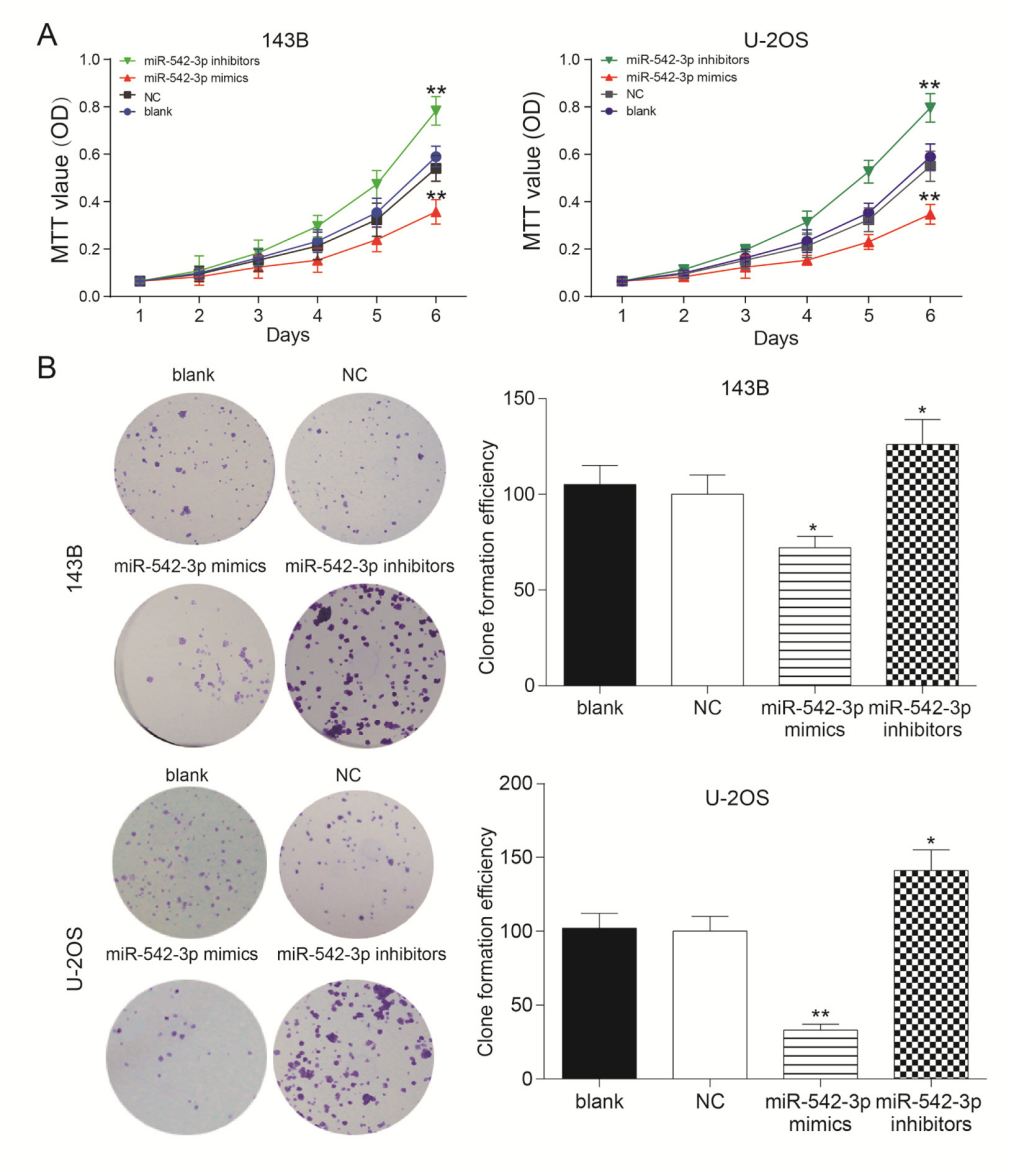
**Overexpression of miR-542-3p suppressed the proliferation of osteosarcoma cells**. (**A**) MTT assay showed that miR-542-3p mimics slowed down the growth of osteosarcoma cell line 143B and U-2OS, while miR-542-3p inhibitors enhanced it. **P<0.01 compared with NC or blank group; (**B**) Colony-forming growth assay, overexpression of miR-542-3p suppressed the proliferation of 143B and U-2OS cells, whereas low expressed miR-542-3p promoted it. *P<0.05, **P<0.01 compared with the NC group.

The growth curve gauged by MTT assay validated that the proliferation rate of miR-542-3p mimics group was prominently lower compared with other groups in U-2OS and 143B cells (*P*<0.01), while the proliferation rate of miR-542-3p inhibitors group increased dramatically compared with NC group (*P*<0.01, Figure 2A). There were no remarkable differences between the blank group and NC group (*P*>0.05, Figure 2A). The results of plate cloning showed that the clones numbers in miR-542-3p mimics group was markedly lower in comparison with NC group in 143B and U-2OS cells (*P*<0.01, Figure 2B), while the cells in miR-542-3p inhibitors group demonstrated significant higher cloning ability compared with NC group (*P*<0.01, Figure 2B). There were no noteworthy differences between the blank group and NC group (*P*>0.05, Figure 2B).

### MiR-542-3p overexpression induced cell apoptosis and cell cycle stagnation in osteosarcoma cells

**Figure 3:**
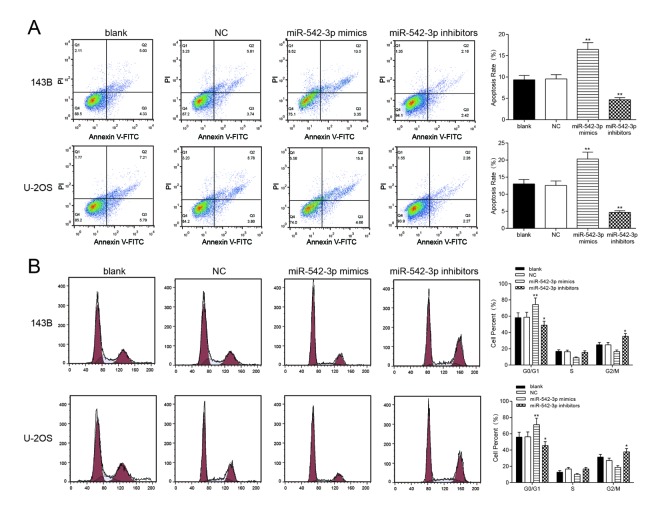
**Overexpression of miR-542-3p induced apoptosis and cell cycle was arrested in osteosarcoma cells**. (**A**) After transfection, the cells were collected and stained with Annexin V-FTIC/PI. The percentages of early (low right quadrant) and late apoptotic cells (upper right quadrant) were assessed by flow cytometry. **P<0.01 compared with the NC group; (**B**) The effect of miR-542-3p on cell cycle progression of 143B and U-2OS cells after 48h incubation was detected by flow cytometry analysis. *P<0.05, **P<0.01 compared with the NC group.

Flow cytometry revealed that the apoptosis rate of miR-542-3p mimics group was greatly higher compared with NC group in osteosarcoma cells, and miR-542-3p suppression could significantly decrease apoptosis rate (*P*<0.01, Figure 3A). Furthermore, cells in the G0/G1 period of the miR-542-3p mimics group accounted for a larger proportion in comparison with NC group, while cells proportion in the G0/G1 period of the miR-542-3p inhibitors group was conspicuously smaller compared with NC group (*P*<0.01, Figure 3B). We concluded that the overexpression of miR-542-3p arrest the cell cycle in G0/G1 period.

### Overexpression of miR-542-3p restrained cell invasion and migration in osteosarcoma cells

**Figure 4:**
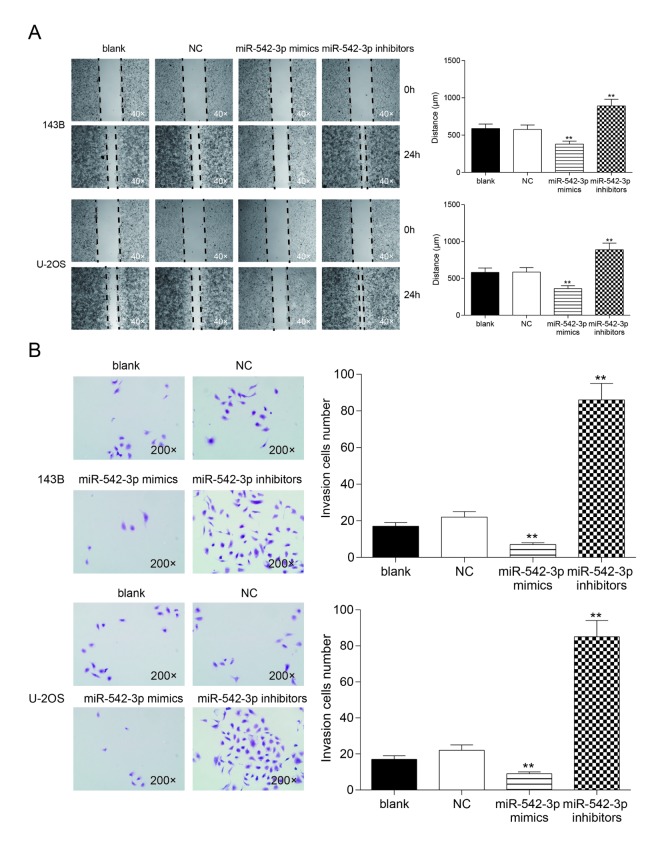
**Overexpression of miR-542-3p suppressed cell migration and invasion in osteosarcoma cells**. (**A**) Wound-healing assay was performed to determine the migratory capacity of 143B and U-2OS cells transfected with water (blank) or scramble miRNA (NC) or miR-542-3p mimics or inhibitors, respectively. **P<0.01 compared with the NC group; (**B**) Transwell assay was performed to determine the invasive capacity of 143B and U-2OS cells transfected with water (blank) or scramble miRNA (NC) or miR-542-3p mimics or inhibitors, respectively. **P<0.01 compared with the NC group.

Wound healing assay revealed that the migrating ability of cells in miR-542-3p mimics group were significantly lower than that in NC group in 143B and U-2OS cells, while cells in inhibitors group demonstrated a better migrating ability compared with NC group (*P*<0.01, Figure 4A). There was no considerable difference between the blank group and NC group (*P*>0.05, Figure 4A). Transwell assay showed that the invasive ability of miR-542-3p mimics group was significantly lower than that in NC group in 143B and U-2OS cells (*P*<0.01), but the number of invasive cells in inhibitors group was noticeably higher compared with NC group (*P*<0.01, Figure 4B). There was no remarkable difference between the blank group and NC group (*P*>0.05, Figure 4B).

### *ILK* is a target of miR-542-3p

**Figure 5:**
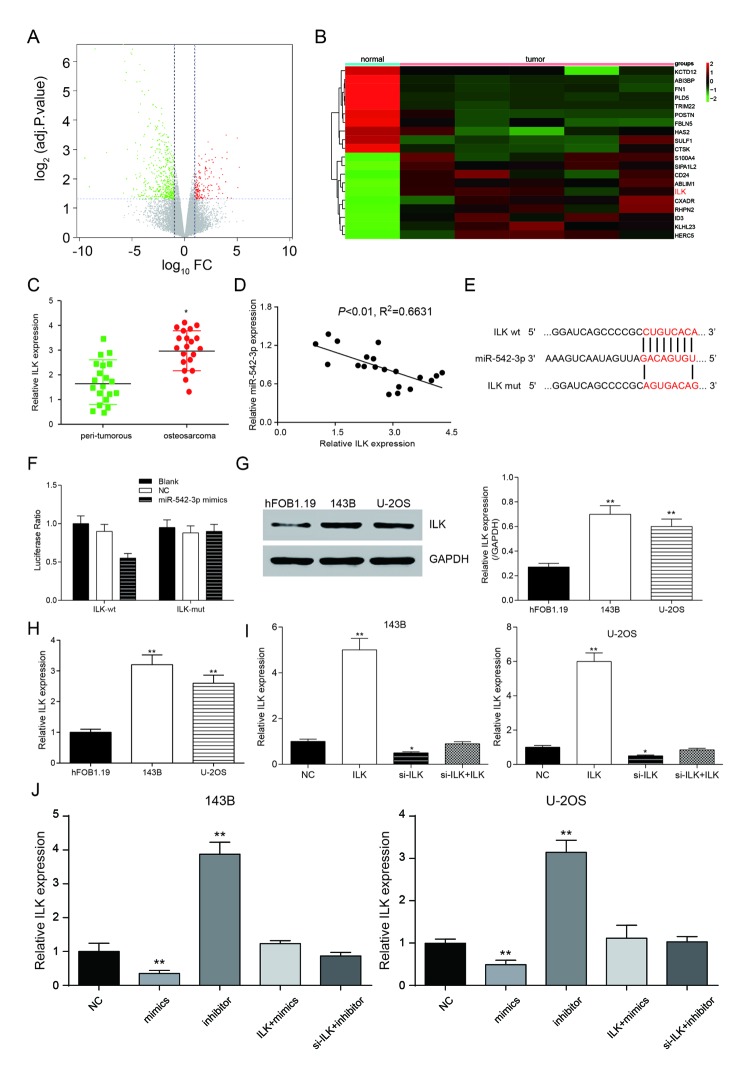
***ILK* is a target of miR-542-3p.** (**A**) Volcano plot showed the variation in gene expression. The negative log of adj.P.Val (base 10) is plotted on the y-axis, and the log of the FC (base 2) is plotted on the x-axis; (**B**) Heat map of differentially expressed mRNAs in normal and osteosarcoma tissues; (**C**) *ILK* expression in osteosarcoma tissues were examined by qRT-PCR. *P<0.05 compared to the peri-tumorous group; (**D**) Scatter diagram exhibited a negative correlation of *ILK* and miR-542-3p in 20 pairs of osteosarcoma tissues by qRT-PCR; (**E**) The binding site in miR-542-3p and 3'-UTR of *ILK* were indicated by TargetScan; (**F**) Luciferase reporter assay data found that co-transfection of osteosarcoma cells with miR-542-3p mimics and wild-type (WT) *ILK* 3'-UTR significantly decrease the luciferase activity, whereas co-transfection with mutant-type (MUT) *ILK* 3'-UTR and miR-542-3p mimics showed no difference with the control group; (**G**) Western blot was used to tested the expression of *ILK* in the normal human osteoblastic cell line hFOB1.19 and the human osteosarcoma cell lines 143B and U-2OS; (**H**) RT-PCR was used to quantify the endogenous levels of *ILK* in hFOB1.19, 143B and U-2OS. **P<0.01 compared with the hFOB1.19 group; (**I**) RT-PCR was used to measure *ILK* expression levels after transfection of a pcDNA3.1-*ILK* and si-*ILK* in 143B and U-2OS cells. *P<0.05, **P<0.01 compared with the NC group. (**J**) *ILK* expression was inhibited by miR-542-3p mimics (mimics group), which reversed by miR-542-3p inhibitor (inhibitor group). **P<0.01 compared with the NC group.

Taking the fold change value exceeding 2 with *P*<0.05 as the screening conditions, the volcanic map was obtained (Figure 5A). Heat map was gained according to the results and *ILK* was up-regulated in human osteosarcoma (Figure 5B). Subsequently, the expression of *ILK* in 20 clinical samples was detected by qRT-PCR. The results showed that *ILK* was up-regulated in osteosarcoma tissue and negatively correlated with miR-152-3p (Figure 5C-5D). TargetScan predicted the binding sites of miR-542-3p and *ILK* (Figure 5E). The dual-luciferase assay showed that the addition of miR-542-3p mimics restrained the activity of luciferase in the wild-type group, suggesting that miR-542-3p could bind to the 3'-UTR seed sequence of *ILK* gene (Figure 5F). The expression of *ILK* in 143B, U-2OS and hFOB.19 was validated by western blot and qRT-PCR. The results showed that *ILK* expression was much higher in osteosarcoma cells in comparison with normal cells (*P*<0.01, Figure 5G-5H). pCDNA3.1-*ILK* and si-*ILK* were transfected into 143B and U-2OS cell lines, and there was a remarkable difference in the *ILK* expression level among the overexpression group, inhibition group and the control group (*P*<0.05, *P*<0.01, Figure 5I). In Figure 5J, the results showed that the expression of *ILK* was down regulated by miR-542-3p mimics (mimics group), which revered by miR-542-3p inhibitor (inhibitor group). The *ILK* level was restored by pcDNA3.1-*ILK* or si-*ILK*. These results between different cell line (143B cell line and U-2OS cell line) were similar.

### Low expression of *ILK* inhibited the proliferation of osteosarcoma cells

**Figure 6:**
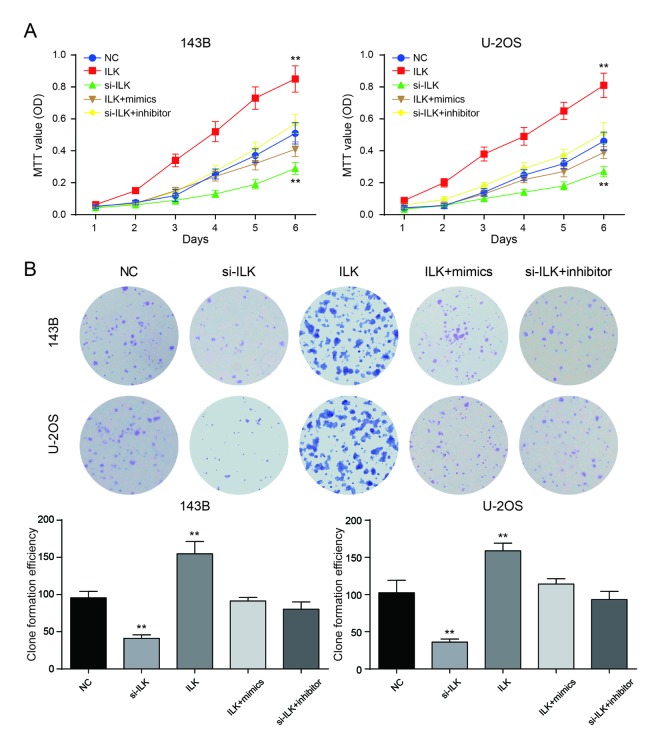
**Low expression of *ILK* inhibited the proliferation of osteosarcoma cells**. (**A**) The MTT assay revealed that overexpression of *ILK* (*ILK* group) promoted proliferation of osteosarcoma cells. **P<0.01 compared with the NC group; (**B**) Colony-forming growth assay was performed to determine the proliferation of pcDNA3.1-*ILK* group (*ILK* group), si-*ILK* group, pcDNA3.1-*ILK*+miR-542-3p mimics group (*ILK*+mimics group) and si-*ILK*+miR-542-3p inhibitor group (si-*ILK*+inhibitor group) transfected osteosarcoma cells. Colonies were counted and captured. **P<0.01 compared with the NC group.

MTT assay showed that *ILK* overexpression could significantly promote cell proliferation in 143B and U-2OS cells (*P*<0.01), while the proliferation rate of si-*ILK* cells was remarkably lower in comparison with NC group (*P*<0.01, Figure 6A). There was no considerable difference between NC group and co-transfection groups (*P*>0.05, Figure 6A). The number of clones in the *ILK* overexpression group was remarkably larger compared with NC group (*P*<0.01), while the clonal ability of NC group was significantly better compared with inhibition group (*P*<0.01, Figure 6B). There was no remarkable difference in the number of cloned cells between NC group and co-transfection groups (*P*>0.05, Figure 6B).

### Low expression of *ILK* promoted apoptosis and arrested cell cycle in osteosarcoma cells.

**Figure 7:**
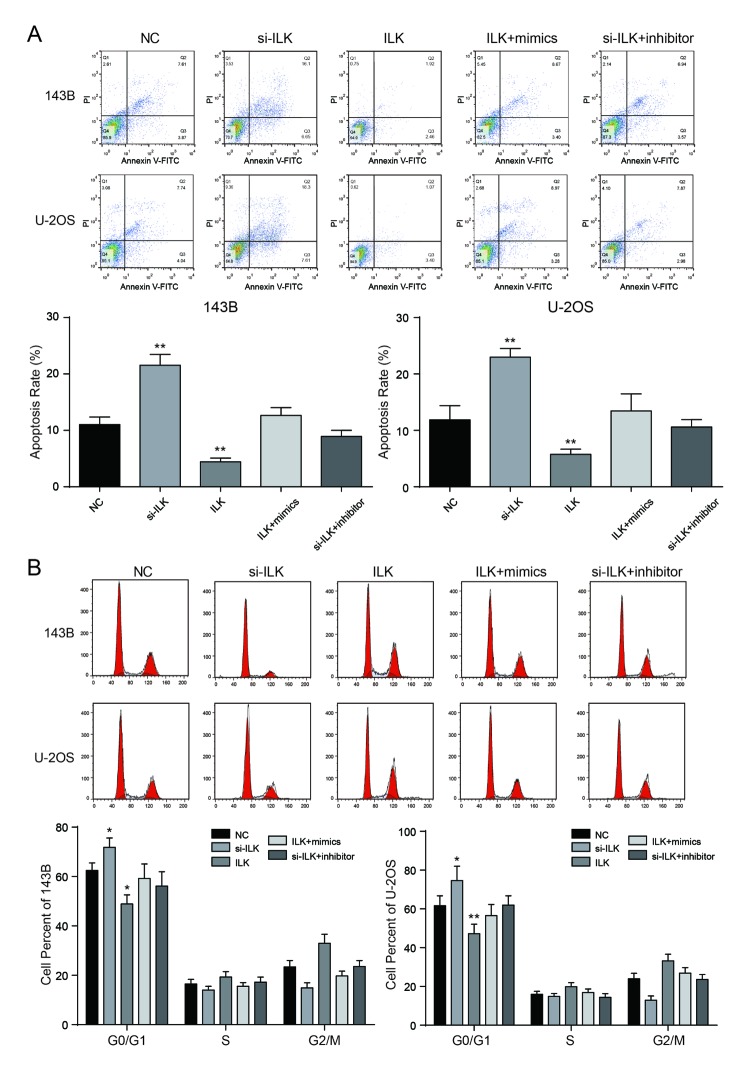
**Low expressed *ILK* promoted apoptosis and arrested cell cycle in osteosarcoma cells**. (**A**) After transfection, the cells were collected and stained with Annexin V-FITC/PI. The percentages of early (low right quadrant) and late apoptotic cells (upper right quadrant) were assessed by flow cytometry. **P<0.01 compared with the NC group; (**B**) The effect of *ILK* on cell cycle progression of 143B and U-2OS cells after 48h incubation was detected by flow cytometry analysis. *P<0.05, **P<0.01 compared with the NC group.

Flow cytometry demonstrated that the apoptosis rate of *ILK* overexpression group was much lower in comparison with NC group in 143B and U-2OS cells, while the apoptosis rate of si-*ILK* group was remarkably augmented (*P*<0.01, Figure 7A). Cells in the G0/G1 period of si-*ILK* group accounted for larger proportion in comparison with NC group (*P*<0.01, Figure 7B), while cells in the G0/G1 period of *ILK* overexpression group accounted for much smaller proportion compared with other groups, which indicated that restraining the expression of *ILK* could stagnate the cell cycle in G0/G1 period and restrain the cell cycle progression. Nevertheless, there was no remarkable difference in apoptosis rate and progression of cell cycle between the co-transfected groups and the control group (*P*>0.05).

### Low expression of *ILK* inhibited cell invasion and migration in osteosarcoma cells

**Figure 8:**
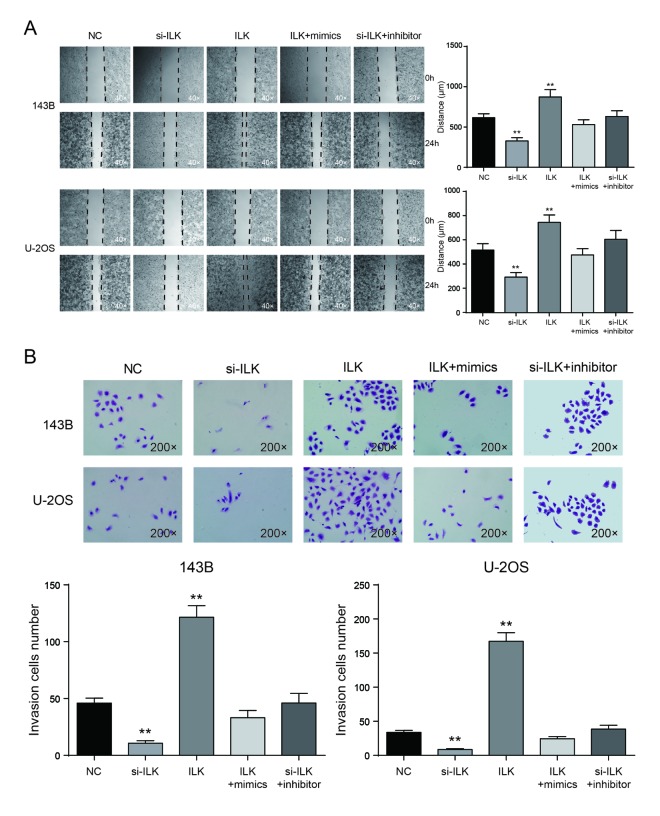
**Low expression of *ILK* inhibited cell migration and invasion in osteosarcoma cells.** (**A**) Wound-healing assay was performed to determine the migratory capacity of 143B and U-2OS cells after transfection, **P<0.01 compared with the NC group; (**B**) Transwell assay was performed to determine the invasive capacity of 143B and U-2OS cells after transfection. **P<0.01 compared with the NC group.

Wound healing assay proved that the migrating ability of *ILK* overexpression group was significantly higher compared with NC group in 143B and U-2OS cells, while the migrating ability of si-*ILK* group was prominently lower compared with NC group (*P*<0.01, Figure 8A). There was no remarkable difference between the co-transfected groups and NC group (*P*>0.05, Figure 8A). Transwell assay showed that in 143B and U-2OS cells, invasive ability of *ILK* overexpression group was much higher than NC group (*P*<0.01), but cells in si-*ILK* group were lesser invasive than NC group (*P*<0.01, Figure 8B). There was no remarkable difference between NC group and co-transfected groups (*P*>0.05, Figure 8B).

### MiR-542-3p overexpression inhibited tumor growth

**Figure 9:**
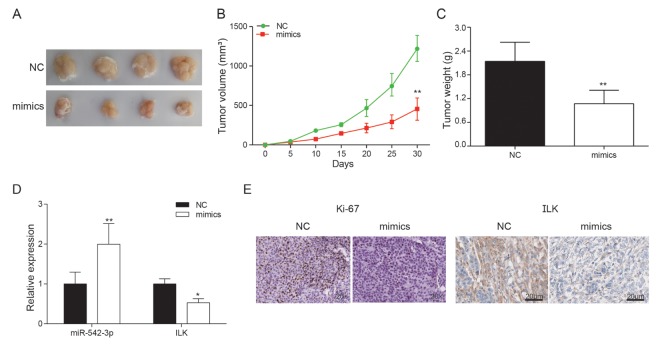
**Effects of miR-542-3p overexpression on tumor growth *in vivo***. (**A**) Tumors were collected from nude mice injected with osteosarcoma cells transfected with miR-542-3p mimics or NC; (**B**) The tumor volume was analyzed every 5 days. **P<0.01 compared with the NC group; (**C**) The tumor weight was measured 30 days after tumor transplantation. **P<0.01 compared with the NC group; (**D**) Expression levels of *ILK* and miR-542-3p were measured by qRT-PCR in nude mice tissues. *P<0.05, **P<0.01 compared with the NC group; (**E**) Expression of ki67 and *ILK* in each group was detected by immunohistochemical staining.

Compared with NC group, miR-542-3p overexpression markedly superseded the growth of subcutaneous tumor (Figure 9A). The tumor volume of mimics group was noticeably smaller in comparison with that of control group (*P*<0.01, Figure 9B). At the 35th day, the nude mice were sacrificed with the tumor removed and weighted. The tumor volume of miR-542-3p overexpression group was much smaller than NC group (*P*<0.01, Figure 9C). The expression of miR-542-3p and *ILK* was detected by qRT-PCR. The results demonstrated that *ILK* was significantly inhibited by miR-542-3p overexpression (*P*<0.01, Figure 9D). Immunohistochemistry revealed that miR-542-3p mimics significantly inhibited *ILK* and ki-67 expression (Figure 9E). *In vivo* experiments demonstrated that miR-542-3p overexpression considerably restrained the proliferation of osteosarcoma cells, which further verified that miR-542-3p suppressed osteosarcoma by targeting *ILK*.

## DISCUSSION

Osteosarcoma, as a primary mesenchymal tumor and one of the most prevalent malignancies, is a common bone tumor in children, accounting for 6% of all childhood cancers [7]. Local invasion and metastasis are the primary reasons for death in osteosarcoma patients [7]. Previous studies have suggested that osteosarcoma was relative higher in metastasis, with 80% of the metastases occurring in lung and the others in bone and lymph nodes [19]. Nowadays, one of the most prevalent therapies for osteosarcoma is pre-and post-operational chemotherapy combined with surgical treatment. Unfortunately, despite advancement in the treatment and diagnosis of osteosarcoma, the total survival rate has been stagnated since the mid-1980s. Common risk factors related to osteosarcoma development include ionizing radiation, Paget’s disease, alkylating agents, the Li-Fraumeni familial cancer syndrome, hereditary retinoblastoma, and other chromosomal abnormalities [2]. In spite of great efforts in investigating the underlying mechanisms in osteosarcoma carcinogenesis, the prognosis for osteosarcoma patients still remain unsatisfied. Therefore, elucidating the molecular mechanism underlying osteosarcoma will contribute to exploring for new diagnostic biomarkers and therapeutic targets for osteosarcoma [20,21]. In our study, we studied the interaction between miR-542-3p and its target gene *ILK*, and further analyzed the influence of miR-542-3p and *ILK* on osteosarcoma.

MiRNAs fulfill their biological function via the regulation of their target genes. Many studies have revealed that miRNAs could inhibit the development of osteosarcoma [22]. During the past decades, miRNAs have been found to be an important regulator in various biological processes such as cell proliferation, metastasis, differentiation, transcriptional regulation and tumorigenesis [3]. Previous studies have shown that the miR-542-3p was substantially down-regulated in the over-expression of c-Src of human cancer cells, and the ectopic expression of miR-542-3p suppressed tumor growth [14]. Kureel et al. have showed that miR-542-3p, an apoptosis inhibitor, reduced mRNA and protein levels of the survival [12]. Study of Cai *et al.* strongly suggested that miR-542-3p down-regulation in astrocytoma promote the expression of crucial positive regulators in AKT signaling, including PI3KR1, and *ILK* [23]. Our study demonstrated that miR-542-3p might be an anti-oncogenic factor in the development of osteosarcoma. According to qRT-PCR, miR-542-3p was down-regulated in osteosarcoma tissues and cells. MTT assay and plate cloning revealed that miR-542-3p suppressed the proliferation of osteosarcoma cells, and miR-542-3p overexpression induced apoptosis, suppressed migration and invasion, and stagnated cell cycle in osteosarcoma cells. Therefore, it was concluded that miR-542-3p overexpression inhibited tumor growth*.*

*ILK,* a highly conserved, 59 kDa serine/threonine kinase, has been involved in controlling various biological processes that are crucial to the progression of malignant disease [16]. *ILK* promotes tumor proliferation and cell-cycle progression in many types of cancer, but its function in osteosarcoma is less clear [24,25]. In our study, the results of dual-luciferase assay revealed the targeting regulatory relations between miR-542-3p and *ILK*, which proved that *ILK* is a target of miR-542-3p. Furthermore, low expression of *ILK* inhibited the proliferation, apoptosis of osteosarcoma cells, as well as promoted cell migration and invasion.

To summarize, through qRT-PCR and western blot, the expression of miR-542-3p was down-regulated, while *ILK* was up-regulated in osteosarcoma tissues and cells. The overexpression of miR-542-3p and silence of *ILK* significantly inhibited proliferation, migration and invasion, which also promoted cell apoptosis and arrested the cell cycle in G0/G1 period. Through our experiments the overexpression of miR-542-3p could down-regulate its target gene *ILK*, thereby promote the apoptosis of osteosarcoma cells and inhibit migration, invasion and proliferation. Overexpression of miR-542-3p could inhibit the growth of osteosarcoma *in vivo*. Despite limitations in our study that we only focused on two cell lines, this research still provides a novel orientation for therapies of osteosarcoma.

## METHODS

### Cells and tissues collection

In this experiment, the osteosarcoma cells 143B, U-2OS and human osteoblasts hFOB1.19 employed were bought from BeNa Culture Collection (Beijing, China). The culture media used for the 143B cells were RMPI1640 media including 10% fetal bovine serum. The media used for U-2OS cells contained 85% RPMI-1640 (# 31800022, GIBCO, Grand Island, NY, USA), 10% heat inactivated horse serum and 5% high quality fetal bovine serum. The two cell lines were cultured at indoor temperature in the cell incubators with 5% CO_2_.

Twenty pairs of human primary osteosarcoma tissues were collected from Huai’An First People’s Hospital. The specimens were frozen immediately in an incubator filled with liquid nitrogen and then reserved at -80°C for analysis. Every patient has consent the prior informed agreement with experimental protocol endorsed by the Ethics Committee of Huai’An First People’s Hospital.

### Bioinformatics analysis

The GSE70414 which included mRNA expression data in five OS cell lines and human mesenchymal stem cells (hMSCs) was analyzed according to Affymetrix HG-U133 plus 2.0 (GPL570) datasets. GSE710414 microarray was downloaded from Gene Expression Omnibus (https://www.ncbi.nlm.nih.gov/geo/). Then, GSE710414 microarray was carried out for difference analysis using Limma R package and the result was visualized by pheatmap R package. The differentially expressed mRNA was selected when fold change value exceeding 2 with *P*<0.05. TargetScan (http://www.targetscan.org/vert_71/) was employed to predict binding sites for miRNAs and mRNAs.

### Cell transfection

MiR-542-3p mimics, miR-542-3p inhibitors, *ILK*-pcDNA3.1 and si-*ILK* were purchase from GenePharma (Shanghai, China). 24h before transfection, U-2OS and 143B cells in the logarithmic growth period were digested by pancreatin and cell suspension was established. Cells were added into 6-well plates and put into incubators at 37°C for 18-24h with 5% CO_2_. 3h before transfection, cells at about 80–90% confluency were changed to the serum and antibiotic-free media. Then, transfection was implemented using Lipofectamine 3000 reagent (Life Technologies, MD, USA) in line with manufacturer’s instructions and incubated at 37°C for 48h with 5% CO_2_. MiR-542-3p mimics, miR-542-3p inhibitor, siRNA labeled by Cy3 were from Sigma (St. Louis, MO). Transfection efficiency was analyzed using ﬂuorescence microscopy (Olympus, Cellsens Dimension).

### Real time quantitative polymerase chain reaction (qRT-PCR)

Total RNA was extracted from tumor tissues and cells were cultured using Trizol reagent (Invitrogen, CA, USA) in line with instructions. NanoDrop 2000 (Thermo Fisher Scientific Inc, MA, USA) was adopted for quantification. Then, 200ng total RNA was employed for reverse transcriptase (Promega, Fitchburg, WI, USA) in light of the instructions of the ReverTra Ace qPCR RT Kit (Toyobo, Japan). The product gained from reverse transcription was then subjected to real-time quantitative PCR analysis using THUNDERBIRD SYBR^®^ QRT Mix (Toyobo, Japan). Conditions for PCR reaction were as follows: pre-denaturation at 94°C for 2 min; denaturation at 94°C for 30s; annealing at 56°C for 30s; extension at 72°C for 1min; 30 cycles; extension at 72°C for 10min. The $2^{-∆∆C_{t}}$ method was employed for quantification of the relative expression levels of miRNA and mRNA. The relative levels of *ILK* were normalized to GAPDH and the relative levels of miR-542-3p were normalized to U6. The experiment was operated three times in every group. Primer sequences employed were presented in Table 1.

**Table 1:** Primer sequences.

**Gene**		**Sequences**
*ILK*	Forward	5’-CTTTGCTGACCTCTCCAATATGG-3’
	Reverse	5’-GGAAATACCTGGTGGGATGGT-3’
miR-542-3p	Forward	5’-GATCATCATGTCACGAGATAC-3’
	Reverse	5’-CTCCCAGACCTTTCAGTTAT-3’
GAPDH	Forward	5’- GACTCATGACCAC AGTCCATGC-3’
	Reverse	5’- AGAGGCAGGGATGATG TTCTG -3’
U6	Forward	5’-CTCGCTTCGGCAGCACATA-3’
	Reverse	5’-AACGATTCACGAATTTGCGT-3’

### MTT assay

Cell suspension was carried out with cells and nutrient solution containing 10% fetal bovine serum. The cells were infused into 96-well plates at a density of 1000-10000 cells per well. At 1^st^, 2^nd^, 3^rd^, 4^th^, 5^th^, 6^th^ day after transfection, 10μl MTT reagent (5mg/ml, prepared with PBS, pH=7.4) was infused to each well and the compound was cultured for 4h. After incubation, 100μl dimethl sulfoxide (DMSO) was added so as to dissolve the crystals. Optical density (OD) value of every sample was measured at a wavelength of 490nm on a Microplate Reader.

### Flow cytometry

After transfection for 72h, cells of the transfection group and control group were digested to obtain cell suspension. After centrifugation, cells were washed and fixed by 75% ethanol at 4°C for 4h, and then flushed with PBS after being centrifuged. Next, 1ml staining solution containing 40μg propidium iodide (PI) and 100μg RNase was infused and cells were incubated for 15min at indoor temperature. Lastly, the cell cycle was detected using a FACS Calibur flow analyzer (BD, CA, USA) and the statistics were subjected to analysis with FACS Diva (BD, USA) software, and the experiment was conducted at least three times.

48h after being transfected, the cells in transfection group and NC group were gathered, washed and resuspended in accordance with the instruction of PI Annexin V Kit (BD, CA, USA). The FACS Calibur was employed to observe the apoptosis and the statistics were subjected to analysis by FACS Diva software. The experiment was operated at least three times.

### Plate clone formation assay

Digested with trypsin solution, the cells were added into RPMI1640 medium. Cell suspension was added into petri dishes at a density of 1×10^3^ per well. Then culture dishes were incubated for 1 to 2 weeks at 37°C in an incubator with 5% CO_2_. Incubation stopped when the cell clones were visible. After removing the supernatant, cells were flushed with PBS and fixed with 4% paraformaldehyde for 15min. After staining with GIMSA for 10-30min, cells were allowed for air dry. Then, cell colony forming units were counted. Assays were conducted at least three times independently.

### Transwell assay

After transfection, 143B and U-2OS cells were subjected to resuspension in serum-free medium and digested with pancreatin. After counting, 200μl cell suspension (containing 5×10^3^ cells) was injected to the top chamber, while 500μl DMEM medium including 10% FBS was infused to the lower chamber Followed by incubating in a moist incubator for 24h at 37°C, cells that invaded through the transwell chamber were washed and fixed with 4% formaldehyde and then dyed with 0.1% crystal violet. The invasion ability of the cells can be detected by a microscope.

### Wound healing assay

Added in 6-well plates at a density of 10^4^ per well, cells were then allowed to reach 90% confluence. A wound was artificially created by scratching the cell monolayer with a 10μl pipette tip. Plates were flushed with PBS to remove the detached cells and cultured in a serum-free medium in an incubator containing 5% CO_2_ at 37°C. Wound healing was detected at 0 and 24^th^ hour, and the image was photographed using a microscope. The average migration distance in each group was calculated.

### Western blot

Total proteins from cells and tissues were extracted with RIPA lysis buffer (Beyotime, Shanghai, China). Quantification for protein concentration was conducted using Pierce BCA Protein Assay Kit (Pierce, Rockford, IL, USA), 100μg proteins were isolated by SDS-PAGE, and then subjected to a polyvinylidene fluoride (PVDF) membrane. Then the membrane was immersed in TBST containing 5% defatted m*ILK* at indoor temperature for 1h and cultured overnight with primary antibodies at 4°C (anti-*ILK*, sc-20019, 1:500, Santa Cruz Biotechnology, CA, USA; anti-GAPDH, ab181603, 1:10000, Abcam, MA, USA). Flushed with TBST for 3 times and added with secondary antibodies (ab7090, 1:2000, Abcam, Cambridge, MA, USA), the membrane was incubated in a shaker at indoor temperature. Flushed with TBST for 3 times, proteins were visualized using ECL-plus reagents (Millipore, Billerica, MA, USA). The Image J software (Version1.48u, Bethesda, USA) was adopted to gauge the density of the bands with GAPDH served as an internal control.

### Dual-luciferase assay

Cells were infused in 24-well plates at a density of $l{\times 10}^{5}$ and incubated before transfection. The 3'-UTR of *ILK* was ligated to the pmiR-RB-REPORORT^TM^ Vector (Ribobio, Guangzhou, China) and the wide-type or mutant-type luciferase reporter plasmids and miR-542-3p mimic were co-transfected. 48h after the transfection, the cells were flushed with PBS. 80μl 1×Passive Lysis Buffer was added into each well. After incubation for 15min at indoor temperature in a shaker, the cells were lysed. Then cell lysis buffer was collected at 4°C and centrifuged for 2min. The supernatant was collected and refrigerate at -80°C refrigerator. Cell lysis buffer was added to 96-well plate with 10μl per well. During the detection, 30 μl luciferase reagents II (Promega, WI, USA) were added. Then 30μl stop buffer were added to terminate the activities. Finally, luciferase activities were recorded.

### Tumor formation in nude mice

Eight female BALB/C nude mice (4 weeks old, purchased from Shanghai SLAC Laboratory Animal Co.,Ltd, China) were used for xenograft tumor growth assay. 200μl osteosarcoma cells transfected with NC and miR-152-3p mimics were injected into the tibial medullary cavity of nude mice. Tumor volumes were calculated by length and width every week (7 days). After 30 days, the mice were sacrificed, and the tumor tissues were fixed in 4% paraformaldehyde, followed by dehydration, paraffin-embedding, and cutting into sections.

### Immunohistochemistry

A certain amount of citrate buffer (pH=6.0) was added to the sections. After being heated by medical microwave for antigen retrieval, the sections were allowed to cool down to indoor temperature. Each section was added with 1 drop of 3% H_2_O_2_ and incubated for 10min at indoor temperature. Then, they were incubated with goat serum for 15min at indoor temperature and the serum was discarded. Consecutive 4-μm-thick sections were analyzed using primary antibodies against ki-67 and anti-*ILK* (1:100,1:500) (anti-ki67, sc-23900; anti-*ILK*, sc-20019, Santa Cruz Biotechnology, CA, USA) and incubated overnight at 4˚C. Flushed with PBS, sections were added with the secondary antibodies (Abcam, Cambridge, MA, USA) which were labeled with horseradish peroxidase and incubated for 30-40min at 4°C. Cells were dyed and flushed with PBS. Cells that were sealed by epoxy resin and anti-quench reagent were subjected to observation under light microscope.

### Statistical analysis

All statistical analyses were implemented with the help of GraphPad Prism 6.0 (Version 6, CA, USA). Data from each group were expressed as mean ± standard error of the mean (SEM). The statistics were analyzed with two-way analysis of variance (ANOVA). The differences were deemed statistically momentous at *P*<0.05.

### Ethics approval and consent to participate

All procedures followed were in accordance with the ethical standards of Huai’An First People’s Hospital and with the Helsinki Declaration of 1964 and later versions. and obtained written informed consents from all the participants.
